# Functional Components, Antioxidant Activity and Hypoglycemic Ability Following Simulated Gastro-Intestinal Digestion of Pigments from Walnut Brown Shell and Green Husk

**DOI:** 10.3390/antiox8120573

**Published:** 2019-11-21

**Authors:** Yanan Sun, Shanshan Li, Fanhang Zeng, Jingyi Qi, Wen Qin, Cui Tan, Qingying Luo, Dingtao Wu, Qing Zhang, Derong Lin, Hong Chen

**Affiliations:** 1 College of Food Science, Sichuan Agricultural University, Yaan 625014, Sichuan, China; sicausunyanan@163.com (Y.S.); calm945@aliyun.com (S.L.); Zeng_9818@163.com (F.Z.); qipeiyan2992@163.com (J.Q.); qinwen@sicau.edu.cn (W.Q.); cherry12112009@163.com (Q.L.); DT_Wu@sicau.edu.cn (D.W.); zhangqing@sicau.edu.cn (Q.Z.); lindr2018@sicau.edu.cn (D.L.); 2 School of Postgraduates, Sichuan Agricultural University, Yaan 625014, Sichuan, China; tancuilove@163.com

**Keywords:** walnut pigment, oxidation resistance, antidiabetics, in vitro simulated digestion

## Abstract

To assess the effects of digestion on the functional components of walnut pigment and their bioactivities, we developed an in vitro model simulating gastro-intestinal digestion. Results showed an increase in the contents of flavonoids and conjugated phenols (with retention rates higher than 100%) in husk pigment after digestion. The lowest of the 2,2′-azino-bis(3-ethylbenzothiazoline-6-sulfonic acid) (ABTS) radical scavenging abilities was reached in the group with the minimum flavonoid content after digestion. Close correlation was observed between free phenol content and total reducing power, as the reducing power among different groups of husk pigment was in consistent with free phenols changes. The inhibitory effect of walnut pigment on α-amylase with/without digestion enzyme was similar. However, shell pigment showed improved inhibitory effect on α-glucosidase activity, with an increased inhibitory rate of 5.42%. In general, the antioxidant activity and hypoglycemic ability of walnut pigment were prone to chemical and enzymatic changes during simulated digestion, which were also related to the alteration of flavonoids and phenols.

## 1. Introduction

Pigments have generally been applied in foodstuffs, because they can provide a brilliant appearance to the products [1]. Given the concern of food nutrition and safety in recent years, natural pigments have drawn more and more attention than their synthetic competitors in the food industry. Natural pigments are mainly distributed in several parts of plants, such as leaves, roots and seeds [2]. Jalapeño peppers have long been treated as a source of constituents with health-promoting function due to the abundant carotenoids and chlorophylls contents [3]. Anthocyanins, the main pigment of red wine, is responsible for wine’s nutraceutical properties and antioxidant ability [4,5]. According to Rabadan, et al. [6], the virgin oil derived from pistachios offers healthy benefits, which are not only attributed to the composition of fatty acid, but also the presence of pigments. Thus, the bioactive function of plant parts can be related to their pigments.

Walnut brown shell and green husk are the by-products of walnut production, and they are widely used in the pharmaceutical and cosmetic industries [7]. The antioxidant potential and antimicrobial activity of walnut (*Juglans regia L.*) green husks have been confirmed in a previous research [8]. In fact, previous studies on walnut husk and shells with regard to antioxidant activity revealed the polyphenolic compounds in these resources [8,9,10,11]. Stampar, Solar, Hudina, Veberic and Colaric [7] reported the existence of thirteen phenolic compounds in the husk. Furthermore, the abundant flavonoids in a walnut shell were also detected [10]. The walnut pigment from these resources may play a key role in their bioactive function, and it is generally accepted that the digestion process affects the structure of bioactive substances, such as the exposure of functional groups on the surface, thus altering the antioxidant capacity [12]. To our knowledge, studies on the changes of the dialysable bioactive compound or bioactive function of walnut pigments are limited, developing an in vitro digestion model. Hence, a model mimicking the physiological conditions in human digestion is required to predict the bioaccessibility of walnut pigments.

On the basis of previous studies, we applied the in vitro gastro-intestinal digestion model of walnut pigment from its brown shell and green husk. The characteristic changes of antioxidant capacity and hypoglycemic activity from the bioactive substances of the pigment were compared throughout the digestion process. The present study was undertaken to explore the changes of walnut pigment on bioactive substances, as well as their bioactive function during digestion, and also to reveal the correlation between these changes and the components of walnut pigment.

## 2. Materials and Methods

### 2.1. Reagents and Plant Materials

The pepsin, pancreatin and taurocholate used during simulated gastrointestinal digestion were purchased from Yuanye Biotechnology (Shanghai, China). Standards (trolox, rutin and gallic acid) and Folin-Ciocalteu reagent were provided by Sigma-Aldrich (Oakville, ON, Dominion of Canada). Hydrochloric acid (HCl), ethanol (C_2_H_5_OH), sodium bicarbonate (NaHCO_3_), sodium chloride (NaCl), sodium acetate (NaOAc), acetic acid (CH_3_COOH), 2,4,6-tri(2-pyridyl)-1,3,5-triazine, iron chloride (FeCl_3_), ferrous chloride (FeCl_2_), methanol (CH_3_OH), 2,2-diphenyl-1-picrylhydrazyl, potassium persulfate (K_2_S_2_0_8_), 2,2′-azino-bis(3-ethylbenzothiazoline-6-sulfonic acid) diammonium salt ((NH_4_)_2_SO_4_), hydrogen disodium phosphate (Na_2_HPO_4_), sodium dihydrogen phosphate (NaH_2_PO_4_), ferrous sulfate (FeSO_4_), salicylic acid (C_7_H_6_O_3_), 30% potassium hydrogen peroxide (KH_2_O_2_), ferrocyanide ([Fe(CN)_6_]^4−^), sodium nitrite (NaNO_2_), aluminum nitrate (Al(NO_3_)_3_), sodium hydroxide (NaOH), sodium carbonate (Na_2_CO_3_) and cellulase were obtained from Aladdin Biochemical Technology (Shanghai, China). Walnuts (*Juglans hopeiensis Hu*) were collected locally from Hanyuan County, Sichuan province, China.

### 2.2. Preparation of Solid Pigment

The cleaned walnut husk and shell were ground (Kunshan Meimei Instrument Co., Ltd., Jiangsu, China) to 40-mesh powder. The extraction method was performed in accordance with the method of Lao and Giusti [13]. Briefly, 10 g of green husk powder was added into a beaker with 700 mL of ultrapure water, and then the mixture was subjected to ultrasonic extraction in an ultrasonic bath (Kunshan Ultrasonic Instrument Co., Jiangsu, China) at 200 W for 30 min. The extraction liquid was centrifuged (Hettich, Germany) at 3000 rpm for 10 min and purified by acid precipitation (2 mL of 36.5% HCl). Finally, the precipitated pigment was freeze-dried (Martin Christ, Germany). 

The extraction of shell pigment was conducted using an ultrasound-assisted enzymatic method, as described in a previous study [14] with modifications. In brief, 5 g of walnut shell powder was mixed with 0.012 g of cellulase in a 125 mL 50% ethanol solution. Then, the solution was adjusted to pH 3 (acidic). After bathing (DAIHAN LABTECH, Korea) in 40 °C water for 1 h and 80 °C water for 5 min, beakers were placed in an ultrasonic cleaning instrument at 200 W and 60 °C for 40 min. 

After centrifugation, the pigment extract was concentrated to a semi-dry state in a rotary evaporator (Laborota 4000, Heidolph, Schwabach, Germany). Finally, a solid walnut brown shell pigment was obtained by lyophilization. 

### 2.3. In Vitro Gastro-Intestinal Digestion

0.05 g of freeze-dried pigment was dissolved in 50 mL of ultrapure water to obtain the initial pigment solution (1 mg/mL). Then, the digestion was simulated in vitro mainly according to the method of Gunathilake, et al. [15], with minor changes. In general, the simulated digestion was divided into two major stages: simulated gastric digestion and intestinal digestion. The 4 mg/mL pepsin solution was prepared using 0.1 mol/L HCl. Pancreatin (2 mg/mL) and taurocholate (12 mg/mL) were prepared using 0.1 mol/L NaHCO_3_ to obtain simulated intestinal fluid.

After the total preparatory work, 50 mL of 9 mg/mL NaCl, 4 mL of 0.1 mol/L HCl and 8 mL of active pepsin solution were added into two conical flasks. Then the pH was adjusted to 2.0–2.5. Each conical flask containing 4 mL of pigment solution (1 mg/mL) was placed into a shaking water bath at 37 °C and 100 rpm for 1 h. Each flask was filtered to obtain the supernatant, which was stored at −20 °C for further analysis, while another continued to the intestinal digestion.

Segments of dialysis bags (cellulose membrane; average flat width; 33 mm, MWCO 12,000 Da, 15 cm) were rinsed with 0.9% NaCl and sealed with clips at one end. During intestinal digestion, dialysis bags containing 8 mL of 9 mg/mL NaCl and 2 mL of 0.5 mol/L NaHCO_3_ were placed into the flasks and continuously agitated at 37 °C (normal human body temperature) and 100 rpm for 45 min. Then, 3.6 mL of simulated intestinal fluid was joined outside of the dialysis bags. The sample solution was adjusted to pH 7.0 (neutral) before incubation in the shaking water bath at 37 °C and 100 rpm for 2 h. Finally, the dialysis bags were taken out, in which the retention liquid was stored at −20 °C for further determination.

In the present study, the groups without enzyme (no enzyme group of shell pigment, NES; no enzyme group of husk pigment, NEH) were set along with the groups with enzyme (enzyme group of shell pigment, ES; enzyme group of husk pigment, EH). The samples were then prepared in parallel with controls: CS, which was the control group of shell pigment, and CH, which was the control group of husk pigment. 

### 2.4. Determination of Flavonoid

Flavonoid content was measured following the procedure described by Tagliazucchi, et al. [16]. Firstly, 500 μL of sample solution was mixed with 30 μL of 5% NaNO_3_ for 6 min. Secondly, 30 μL of 10% Al(NO_3_)_3_ was added into the solution. After 6 min, the solution was mixed with 400 μL of 4% NaOH for 12 min. Finally, the absorbance was measured at 510 nm by using a UV/VIS spectrophotometer (Optima, SP-3000, Tokyo, Japan). Results were obtained from the standard curve constructed using rutin and expressed in mg rutin equivalents (RE) per milliliter (mg RE/mL). All samples were analyzed in triplicate.

### 2.5. Determination of Phenol

Phenol content was estimated using the Folin-Ciocalteu method of Li, et al. [17]. Generally, 0.5 mL of the sample solution was mixed with 0.5 mL of 0.25 mol/L Folin reagent and allowed to stand for 3 min. Then, 1 mL of 15% Na_2_CO_3_ solution was added. After 30 min, the absorbance was measured at 760 nm using a UV/VIS spectrophotometer. Results were obtained from the standard curve constructed by gallic acid and expressed in mg gallic acid equivalents (GAE) per milliliter (mg GAE/mL). All samples were analyzed in triplicate.

### 2.6. Extraction of Conjugated Phenols

Conjugated phenols were extracted following the acid hydrolysis assay of Ahmad, et al. [18] with some modifications. Briefly, 9 mL of ultrapure water and 1 mL sample solution were mixed in a centrifuge tube. Then 5 mL of 6 mol/L HCl was slowly added into the mixture at a flow rate of 1 mL/min. All of the tubes were placed into a water bath and continuously oscillated at 35 °C for 16 h. Finally, the solution was cooled to ambient temperature and extracted with ethyl acetate at a volume ratio of 3:1, three times. Then anhydrous MgSO_4_ was applied to dehydrate the ethyl acetate extract.

### 2.7. Estimation of the In Vitro Antioxidant Capacity

Five assays were carried out to estimate the in vitro antioxidant capacity of the bioactive substances. Results were acquired from the standard curve constructed using Trolox and expressed in μmol Trolox equivalents (TE) per liter (μmol TE/L). All samples were analyzed in triplicate.

#### 2.7.1. ABTS Radical Scavenging Capacity Assay (ABTS)

The 2,2′-azino-bis(3-ethylbenzothiazoline-6-sulfonic acid) (ABTS) radical scavenging capacity assay was carried out according to Zhang, et al. [19] with some modifications. Briefly, 10 μL of sample liquid was mixed with 200 μL of ABTS^`+^ for 10 min, then the absorbance value was measured at 515 nm in a dark room.

#### 2.7.2. Ferric-Reducing Antioxidant Power Assay (FRAP)

The Ferric-Reducing Antioxidant Power Assay (FRAP) was determined according to a method of Zhang, et al. [20] with some modifications. The entire experiment was carried out at room temperature and in a dark environment. After the reaction of 100 μL of sample solution with 3 mL of FRAP reagent for 6 min, the absorbance value was determined at 593 nm.

#### 2.7.3. Total Reducing Power (RP) Assay 

The RP assay was carried out by modifying the method of Zhang, et al. [19]. In brief, 200 μL of sample solution was mixed with 200 μL of 0.2 M sodium phosphate buffer (pH 6.6, acidic) and 200 μL of 1% (*w*/*v*) K_3_[Fe(CN)_6_]. After incubation at 50 °C for 20 min, the solution was mixed with 200 μL of 10% (*w*/*v*) trichloroacetic acid (TCA) and centrifuged at 400 rpm for 10 min. Subsequently, 200 μL of supernatant was blent with 200 μL of ultrapure water and 4 μL of 0.1% (*w*/*v*) FeCl_3_. Absorbance at 700 nm was recorded.

#### 2.7.4. DPPH Radical Scavenging Capacity Assay (DPPH)

The assay developed by Zhang et al. [19] was employed in this study with modifications. In brief, 100 μL of sample solution was added in 100 μL of 60 μM 2,2-diphenyl-1-picrylhydrazyl (DPPH) (dissolved in methanol). The solution was allowed to react for 30 min at room temperature and dark environment, and the absorbance was measured at 517 nm.

#### 2.7.5. OH Radical Scavenging Activity (OH)

The assay to measure the ^•^OH radical scavenging ability was performed according to the procedure described by Liu, et al. [21]. Briefly, 200 μL sample solution was mixed with 50 μL of 9 mmol/L salicylic acid reagent, 50 μL of 9 mmol/L FeSO_4_ and 650 μL of ultrapure water. The solution was homogenously mixed, combined with 50 μL of 0.03% H_2_O_2_, and then incubated at 37 °C for 30 min. Absorbance at 510 nm was measured.

### 2.8. Estimation of Hypoglycemic Ability

#### 2.8.1. α-Amylase Inhibitory Activity

α-Amylase inhibitory activity was determined following a previously reported method [22]. In brief, 500 μL of sample solution and 500 μL of α-amylase solution (5 mU/mL) was mixed and incubated at 37 °C for 10 min. Then 500 μL of 1% starch solution was added into the mixture and incubated at 37 °C for 10 min. 1 mL of 3,5-dinitrosalicylic acid (DNS) reagent was joined to stop the reaction. Finally, all of the tubes were bathed in boiling water to inactivate the α-amylase. The solution was cooled to room temperature and diluted with 10 mL ultrapure water. Absorbance at 520 nm was measured. The α-amylase inhibitory rate was calculated as follows:

Inhibitory rate (%) = 100 − (A_s_ − A_b_)/A_0_ × 100 (1)

A_s_: The absorbance of the sample solution

A_b_: The absorbance of the reagent without α-amylase

A_0_: The absorbance of the reagent without sample

#### 2.8.2. α-Glucosidase Inhibitory Activity

The method of Chapdelaine et al. [23] was followed to measure the α-glucosidase inhibitory activity. In brief, 50 μL of sample solution was mixed with 100 μL of α-glucosidase solution (0.35 U/mL), and the mixture was incubated at 37 °C for 10 min. The mixture was joined with 100 μL of *p*-nitrophenyl-α-D-glucopyranoside solution (1.5 mM). After 20 min of incubation, 1 mL of 1M Na_2_CO_3_ was added. Absorbance at 400 nm was measured. The α-glucosidase inhibitory rate was calculated as follows:

Inhibitory rate (%) = 100 − (A_s_ − A_b_)/A_0_ × 100 (2)

A_s_: The absorbance of the sample solution

A_b_: The absorbance of the reagent withoutα-glucosidase

A_0_: The absorbance of the reagent without sample

### 2.9. Statistical Analysis

All statistical analyses were performed using the SPSS 17.0 statistical package (SPSS, Inc., Chicago, IL, USA). Results were presented as mean values ± standard deviation (SD) and analyzed for differences between means by one-way Analysis of Variance (ANOVA) with Tukey’s HSD post hoc test. Significance was accepted at *p* < 0.05.

## 3. Results and Discussion

### 3.1. Comparative Analysis of Flavonoid Retention 

**Figure 1:**
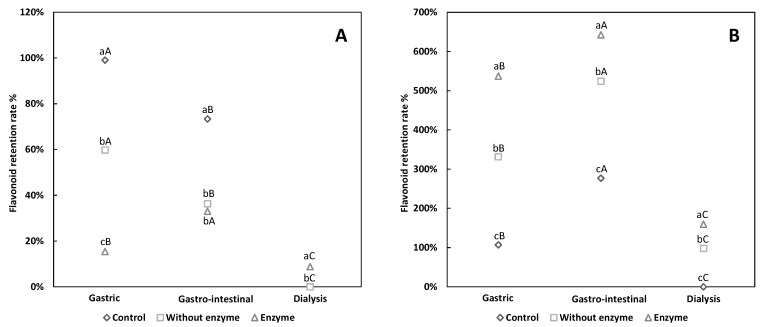
The flavonoid retention rate of walnut pigment from brown shell (**A**) and green husk (**B**) subjected to simulated in vitro gastric and intestinal digestion and dialysis (potential uptake). The data presented in this figure consist of average quantities ± standard deviation (SD) of three independent samples. Different letters in the bars within each group represent statistically significant differences (*p* < 0.05). “a–c” indicates a significant difference in longitudinal, and “A–C” represents a significant difference in lateral.

As is shown in Figure 1, after gastro-intestinal digestion, the flavonoid retention rate of ES and EH was higher than that after the gastric stage. For husk pigment, the flavonoid retention rate of EH and NEH was beyond 100% (Figure 1B) after gastric digestion, signifying that the flavonoid content of husk pigment increased after digestion, and this increase continued to occur until the intestinal digestion phase. Huang, et al. [24] simulated the in vitro digestion of native Royle seed and also observed increased flavonoid content after digestion. They concluded that the alteration of flavonoid content after digestion was related to plant species and phenolic substance composition. For example, anthocyanin, the most abundant flavonoid compound in blueberries, is susceptible to an alkaline environment and thus contributes to the substantial losses of flavonoid during intestinal digestion [25]. Pepsin digestion does not affect the stability of flavonoid during gastric digestion [26]. However, a low pH around 2.0 (highly alkali) could destroy some low-molecular-weight polyphenols [27]. 

This partially explains the decreased retention rate of flavonoid in the shell pigment (Figure 1A). Given that previous studies have not detected anthocyanin in walnut green husk [28], we assumed that the high retention rate of flavonoids in husk pigment was due to high-molecular-weight flavonoids.

At different digestion phases and in the dialysis bags, the flavonoid retention rate was characterized as follows: EH > NEH > CH, which demonstrated that the enzyme along with acid or alkaline substances positively influenced flavonoid release and absorption, with the enzyme showing superior effect.

During gastric digestion, ES reached the lowest retention rate, followed by NES (Figure 1A). The flavonoid retention rate of ES was shown to rise after intestinal digestion, which may be due to the long digestive time. This escalation may be associated with the presence of the flavonoid glycoside form, which shows increased resistance to acid hydrolysis compared with its original form, thus additional content reaches the intestinal digestion phase [29]. Furthermore, the change in the polyphenols structure is also suggested to be due to pH alteration [30].

### 3.2. Comparative Analysis of Free and Conjugated Phenols Retention 

**Figure 2:**
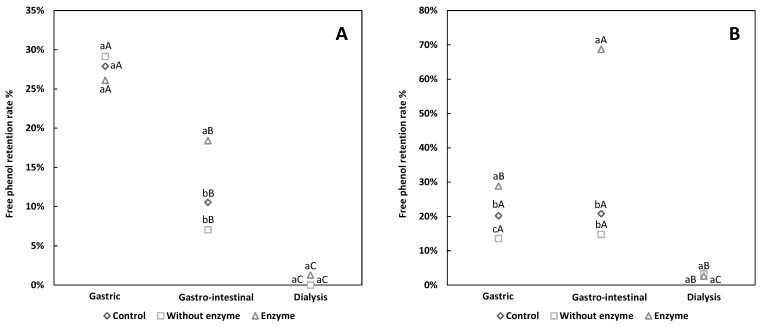
The free phenol retention rate of walnut pigment from brown shell (**A**) and green husk (**B**) subjected to simulated in vitro gastric and intestinal digestion and dialysis (potential uptake). The data presented in this figure consist of average quantities ± SD of three independent samples. Different letters in the bars within each group represent statistically significant differences (*p* < 0.05). “a–c” indicates significant difference in longitudinal, and “A–C” represents significant difference in lateral.

Figure 2A shows that the free phenol retention rate of shell pigment diminished amongst all groups and the retention rate was similar. During gastro-intestinal digestion, the free phenol retention rate of ES was significantly higher than that of NES and CS, and this finding was in agreement with a previous report [31], showing that samples treated with active digestive enzymes had higher total phenolic contents compared with their inactivated-enzyme-treated counterparts. These results implied that the higher phenols in the enzyme group may be due to the protective effect from alkaline substances and/or pancreatin to some extent. 

**Figure 3:**
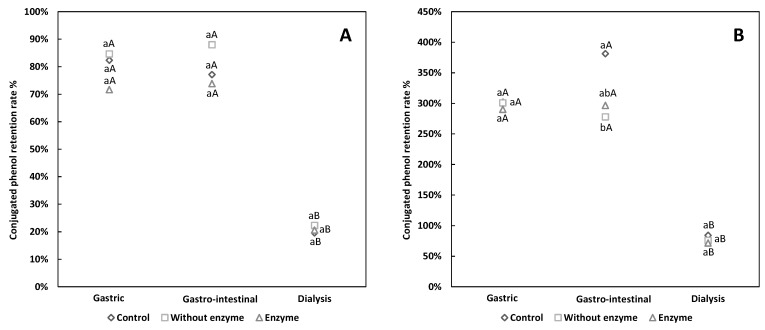
The conjugated phenol retention rate of walnut pigment from brown shell (**A**) and green husk (**B**) subjected to simulated in vitro gastric and intestinal digestion and dialysis (potential uptake). The data presented in this figure consist of average quantities ± SD of three independent samples. Different letters in the bars within each group represent statistically significant differences (*p* < 0.05). “a–c” indicates significant difference in longitudinal, and “A–C” represents significant difference in lateral.

The pattern was similar for husk pigment, as the free phenols in all groups decreased (Figure 2B). The conjugated phenols in husk pigments showed increased content during digestion (Figure 3B), which may be due to the conversion of some free phenols into conjugated phenols. As previous studies confirmed [32], the biologically-active substances could be conjugated to the large molecules (such as water-soluble dietary fiber) in walnut green husk pigment during the digestive process. During gastric digestion, a pectin-phenol mixture may form, which may also be responsible for the reduced free phenol content [27]. Other research reported that the interaction between phenols and fiber is attributed to hydrogen bonds including Van der Waals forces, and non-covalent bonds including electrostatic forces [33], thus affecting the antioxidant capacity of bioactive substances. The free phenol retention rate of EH increased during intestinal digestion, whereas the other groups showed no change (Figure 2B). We consequently deem that the re-release of free phenols may be related to pancreatin, which may help the release of phenolic acids like ellagic acid (one of the major phenolic compounds present in walnut green husk [7]) from the ester form. Moreover, the proportion of free phenols and conjugated phenols in pigment may account for the slight recovery of free phenols during intestinal digestion [15]. Free phenols in the shell pigment decreased during gastro-intestinal digestion. According to a previous study on the simulated digestion of hawthorn, some new unknown peaks were detected by high-performance liquid chromatography (HPLC) after digestion, evidencing the existence of new compounds [34]. Hence, the decreased free phenols in shell pigment maybe converted or degraded into other chemical compounds.

For both of the pigments, the conjugated phenols content of gastric digesta and intestinal digesta were similar. Thus, conjugated phenols may have resistance to pancreatin and/or alkaline conditions.

### 3.3. Digestion Effect on the Antioxidant Ability of Pigments 

The ABTS radical scavenging ability of walnut green husk and brown shell pigment in simulated digestion was shown in Table 1. In the gastric and gastro-intestinal phases, EH showed lower ABTS radical scavenging ability compared with NEH and CH. Walnut shell pigment showed a pattern similar to that of husk pigment during gastric digestion. However, after intestinal digestion, a trend of NES > CS > ES was observed. This finding indicates that the incorporation of pancreatin and alkaline substances can inhibit the ABTS radical scavenging ability of shell pigment. We also speculated that the ABTS radical scavenging ability was mainly related to flavonoid, given that ES reached the lowest flavonoid content compared with the other groups during gastro-intestinal digestion. 

The FRAP of pigments from shell and husk were quite different. During gastro-intestinal digestion, CH reached the highest value, followed by NEH (Table 2). ES had the lowest FRAP value with the presence of pepsin in gastric digestion. Hence, the alkaline substances might inhibit the FRAP of husk pigment, and pancreatin enhanced this inhibitory effect.

The RP of walnut green husk pigment was ordered as EH > CH > NEH during gastric digestion. During intestinal digestion, EH was higher than the other groups (Table 3). The RP of walnut husk pigment during simulated digestion may be related to the free phenols, for the consistency between free phenols changes and RP (Figure 2B). For shell pigment, the relation between RP and free phenols was not as close as that found in husk pigment, indicating the RP of shell pigment may have sensitivity toward pepsin and resistance toward pancreatin. 

The DPPH· scavenging activities of walnut green husk pigment amongst different groups were similar (Table 4). With regard to shell pigment, ES reached the lowest scavenging activity during gastro-intestinal digestion, which was in agreement with flavonoid changes during intestinal digestion. Once DPPH· is scavenged, samples often show a strong ability to scavenge hydroxyl radicals, alkyl radicals and peroxide radicals. As a consequence, the DPPH assay is of good reference for antioxidant testing on the basis of DPPH· reduction [35]. The propensity of flavonoid to scavenge free-radicals is dominated by its chemical structure [36], such as the 2–3 double bond, and OH with an ortho position in C3′–C4′ or OH in C_3_ [10]. Thus, the flavonoid in shell pigment has the above-mentioned structure.

The ·OH scavenging activity of walnut pigment in simulated digestion is shown in Table 5. During gastric digestion, CS and NES reached similar values, which were higher than that of ES. Both pigments without enzyme reached the highest ·OH scavenging activity during gastro-intestinal digestion, which may due to the effect of phenols. Phenols can improve antioxidant activity by converting the hydroxyl groups on the aromatic ring to protons when transferred from acid gastric juice to an alkaline environment [37]. In addition, previous study summarized that free phenols may serve as excellent contributors to antioxidant activity, in contrast with bound ones in teff [38]. However, the ·OH scavenging activity of soluble conjugate and insoluble-bound phenolic fractions is weak [32]. Thus, the ·OH scavenging power detected in the digestive juice in this study may be mainly attributed to free phenols. 

### 3.4. Effect of Digestion on the Hypoglycemic Ability of Pigments

**Figure 4:**
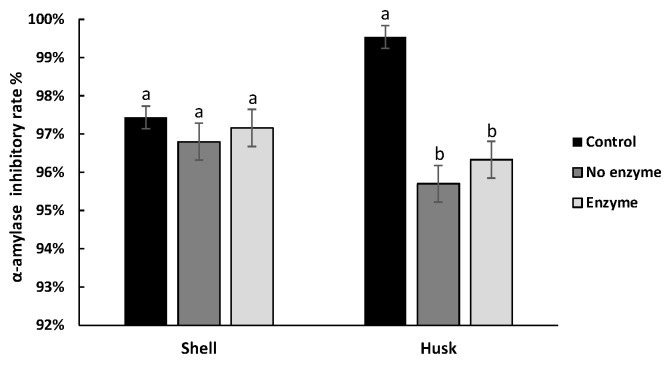
The inhibitory rate of walnut pigment from brown shell and green husk on α-amylase activity with/without enzyme digestion. The data presented in this table consist of average quantities ± SD of three independent samples. Different letters (a–c) in the bars within each group represent statistically significant differences (*p* < 0.05).

**Figure 5:**
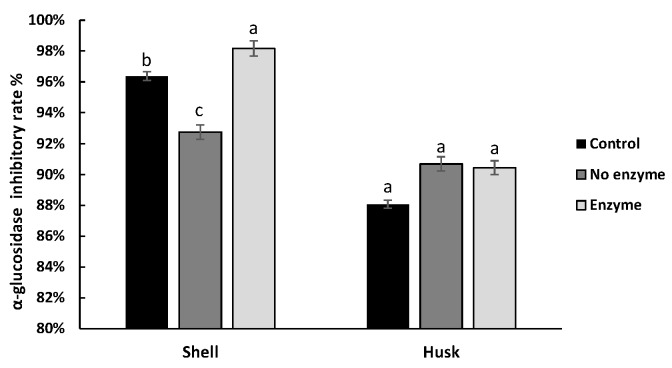
The inhibitory rate of walnut pigment from brown shell and green husk on α-glucosidase activity with/without enzyme digestion. The data presented in this table consist of average quantities± SD of three independent samples. Different letters (a–c) in the bars within each group represent statistically significant differences (*p* < 0.05).

The inhibitory effect of both pigments on α-amylase activity with/without enzyme digestion is shown in Figure 4. For husk pigment, no significant difference between the NEH and EH was found. The same result was observed for NES and ES, indicating that the digestion of enzymes had no significant effect on the inhibition of α-amylase activity of the two pigments. But it is interesting to notice that the α-amylase inhibitory rate of CH could be higher than NEH and EH, which indicated that the digestion environment may impair the α-amylase inhibitory effect of the husk pigment. The inhibition rate of α-glucosidase activity in shell pigment was increased after digestion with enzyme, whereas that of husk pigment changed indistinctively (Figure 5). 

The inhibitory effect of both pigments was well preserved during the simulated digestion. Furthermore, the existence of digestive enzyme was beneficial to shell pigments for their exertion of inhibition ability on α-glucosidase, and with enzyme digestion, the pronouncedly increased α-glucosidase inhibition and the mildly changed effect on α-amylase of shell pigment seem to be favorable in therapy for postprandial hyperglycemia, reducing side effects [39]. 

The α-amylase inhibitors can delay the hydrolysis of starch during small intestine digestion, contributing to a reduction in glucose absorption, thus decreasing the postprandial plasma glucose levels [40]. According to a previous report [41], polyphenols can inhibit α-amylase activity. Consequently, the stable inhibitory rate of green husk pigment to α-amylase may be related to the increased conjugated polyphenols. During digestion, conjugated polyphenols may bind to the enzyme molecules, affecting the hydrolysis toward glycosidic bonds by amylase [42]. Moreover, the α-amylase activity could be affected by condensed (ellagitannins) and hydrolysable tannins (proanthocyanidins) [43]. A previous report [7] revealed the existence of ellagic acids in walnut husk, and this may also explain the α-amylase inhibitory effect in husk pigment. Since the amount and position of hydroxyl groups in flavonoids regulate their α-amylase inhibitory ability [34], flavonoids in husk pigment may undergo structural changes during digestion. In the light of a previous study [44], the inhibition toward the α-glucosidase activity of raspberry dried seeds was improved in simulated small intestinal digestion, suggesting that the digestive progress is beneficial for phenolic compounds to play a hypoglycemic function.

## 4. Conclusions

The simulated digestion may reduce free phenols and flavonoids in shell pigments and increase the content of flavonoids and conjugated phenols in husk pigment. The antioxidant activity and hypoglycemic ability of walnut pigment were prone to chemical and enzymatic changes during simulated digestion, which were also related to the alteration of flavonoids and phenols. With regard to the bioavailability of pigments, walnut green husk pigment may be superior to brown shell, in terms of its better absorption of the bioactive components. 

## Figures and Tables

**Table 1:** The 2,2′-azino-bis(3-ethylbenzothiazoline-6-sulfonic acid) (ABTS) radical scavenging ability of walnut green husk and brown shell pigment subjected to simulated in vitro digestion and dialysis (μmol TE/L).

Groups	Control	Without Enzyme	Enzyme
Walnut green husk pigment			
Gastric	1174.1 ± 14.7 ^a^	1192.4 ± 12.9 ^a^	102.8 ± 1.4 ^b^
Gastro-intestinal	1159.7 ± 34.0 ^a^	1191.1 ± 33.9 ^a^	449.8 ± 31.6 ^b^
Dialysis	349.8 ± 19.2 ^a^	46.2 ± 3.2 ^b^	44.9 ± 1.4 ^b^
Walnut brown shell pigment			
Gastric	910.3 ± 49.5 ^a^	857.6 ± 52.7 ^a^	44.4 ± 3.6 ^b^
Gastro-intestinal	828.2 ± 22.9 ^b^	949.7 ± 49.1 ^a^	311.0 ± 18.5 ^c^
Dialysis	453.8 ± 34.0 ^a^	433.9 ± 9.4 ^a^	250.6 ± 10.3 ^b^

The data presented in this table consist of average quantities ± SD of three independent samples. Different letters (a–c) in rows represent statistically significant differences (*p* < 0.05). NES, no enzyme group of shell pigment; NEH, no enzyme group of husk pigment; ES, enzyme group of shell pigment; EH, enzyme group of husk pigment; CS, control group of shell pigment; CH, control group of husk pigment.

**Table 2:** The ferric-reducing antioxidant power (FRAP) of walnut green husk and brown shell pigment subjecting to simulated in vitro digestion and dialysis (μmol TE/L).

Groups	Control	Without Enzyme	Enzyme
Walnut green husk pigment			
Gastric	71.9 ± 3.1 ^a^	38.9 ± 2.9 ^b^	68.9 ± 3.4 ^a^
Gastro-intestinal	50.6 ± 1.1 ^a^	39.5 ± 2.1 ^b^	31.5 ± 2.8 ^c^
Dialysis	58.3 ± 3.7 ^a^	43.5 ± 1.7 ^b^	42.7 ± 3.1 ^b^
Walnut brown shell pigment			
Gastric	124.1 ± 10.4 ^a^	128.0 ± 7.7 ^a^	83.7 ± 0.6 ^b^
Gastro-intestinal	74.5 ± 3.4 ^a^	55.7 ± 1.6 ^b^	76.2 ± 1.7 ^a^
Dialysis	31.1 ± 2.4 ^a^	16.1 ± 0.4 ^b^	34.6 ± 3.2 ^a^

The data presented in this table consist of average quantities ± SD of three independent samples. Different letters (a–c) in rows represent statistically significant differences (*p* < 0.05). NES, no enzyme group of shell pigment; NEH, no enzyme group of husk pigment; ES, enzyme group of shell pigment; EH, enzyme group of husk pigment; CS, control group of shell pigment; CH, control group of husk pigment.

**Table 3:** The total reducing power (RP) of walnut green husk and brown shell pigment subjected to simulated in vitro digestion and dialysis (μmol TE/L).

Groups	Control	Without Enzyme	Enzyme
Walnut green husk pigment			
Gastric	48.33 ± 0.17 ^b^	44.93 ± 0.18 ^c^	53.03 ± 1.43 ^a^
Gastro-intestinal	38.65 ± 0.25 ^b^	39.74 ± 0.44 ^b^	50.29 ± 2.73 ^a^
Dialysis	29 ± 1.47 ^b^	30.67 ± 0.72 ^b^	35.44 ± 2.00 ^a^
Walnut brown shell pigment			
Gastric	94.41 ± 1.46 ^a^	92.95 ± 0.76 ^a^	84.71 ± 1.57 ^b^
Gastro-intestinal	60.97 ± 2.00 ^b^	53.84 ± 0.89 ^c^	76.86 ± 1.86 ^a^
Dialysis	31.27 ± 0.36 ^b^	30.57 ± 1.12 ^b^	34.34 ± 0.30 ^a^

The data presented in this table consist of average quantities ± SD of three independent samples. Different letters (a–c) in rows represent statistically significant differences (*p* < 0.05). NES, no enzyme group of shell pigment; NEH, no enzyme group of husk pigment; ES, enzyme group of shell pigment; EH, enzyme group of husk pigment; CS, control group of shell pigment; CH, control group of husk pigment.

**Table 4:** The DPPH scavenging activity of walnut green husk and brown shell pigment subjected to simulated in vitro digestion and dialysis (μmol TE/L).

Groups	Control	Without Enzyme	Enzyme
Walnut green husk pigment			
Gastric	838 ± 36 ^a^	944 ± 42 ^a^	920 ± 22 ^a^
Gastro-intestinal	988 ± 25 ^a^	908 ± 19 ^a^	982 ± 27 ^a^
Dialysis	867 ± 17 ^a^	642 ± 24 ^b^	555 ± 12 ^c^
Walnut brown shell pigment			
Gastric	1047 ± 6 ^a^	938 ± 24 ^b^	996 ± 59 ^a,b^
Gastro-intestinal	970 ± 25 ^a^	910 ± 34 ^a^	813 ± 32 ^b^
Dialysis	991 ± 47 ^a^	997 ± 51 ^a^	997 ± 51 ^a^

The data presented in this table consist of average quantities ± SD of three independent samples. Different letters (a–c) in rows represent statistically significant differences (*p* < 0.05). NES, no enzyme group of shell pigment; NEH, no enzyme group of husk pigment; ES, enzyme group of shell pigment; EH, enzyme group of husk pigment; CS, control group of shell pigment; CH, control group of husk pigment.

**Table 5:** The ·OH scavenging activity of walnut green husk and brown shell pigment subjected to simulated in vitro digestion and dialysis (μmol TE/L).

Groups	Control	No enzyme	Enzyme
Walnut green husk pigment			
Gastric	2016 ± 8 ^a^	2006 ± 8 ^a^	1992 ± 6 ^b^
Gastro-intestinal	1726 ± 5 ^b^	1865 ± 7 ^a^	1728 ± 1 ^b^
Dialysis	1897 ± 6 ^b^	2002 ± 8 ^a^	1894 ± 9 ^b^
Walnut brown shell pigment			
Gastric	2032 ± 11 ^a^	2009 ± 6 ^b^	2015 ± 9 ^a,b^
Gastro-intestinal	1744 ± 4 ^b^	1773 ± 12 ^a^	1758 ± 9 ^a,b^
Dialysis	1840 ± 11 ^c^	1893 ± 2 ^b^	1933 ± 8 ^a^

The data presented in this table consist of average quantities ± SD of three independent samples. Different letters (a–c) in the rows represent statistically significant differences (*p* < 0.05). NES, no enzyme group of shell pigment; NEH, no enzyme group of husk pigment; ES, enzyme group of shell pigment; EH, enzyme group of husk pigment; CS, control group of shell pigment; CH, control group of husk pigment.

## References

[1] Parmar R.S., Singh C. (2018). A comprehensive study of eco-friendly natural pigment and its applications. Biochem. Biophys. Rep..

[2] Cervantes-Paz B., Yahia E.M., Ornelas-Paz J.D.J., Gardea-Béjar A.A., Ibarra-Junquera V., Pérez-Martínez J.D. (2012). Effect of Heat Processing on the Profile of Pigments and Antioxidant Capacity of Green and Red Jalapeño Peppers. J. Agric. Food Chem..

[3] Alvarez-Parrilla E., De La Rosa L.A., Amarowicz R., Shahidi F. (2012). Protective effect of fresh and processed Jalapeño and Serrano peppers against food lipid and human LDL cholesterol oxidation. Food Chem..

[4] Favre G., Hermosín-Gutiérrez I., Piccardo D., Gómez-Alonso S., González-Neves G. (2019). Selectivity of pigments extraction from grapes and their partial retention in the pomace during red-winemaking. Food Chem..

[5] Lingua M.S., Wunderlin D.A., Baroni M.V. (2018). Effect of simulated digestion on the phenolic components of red grapes and their corresponding wines. J. Funct. Foods.

[6] Rabadan A., Gallardo-Guerrero L., Gandul-Rojas B., Álvarez-Ortí M., Pardo J.E. (2018). Effect of roasting conditions on pigment composition and some quality parameters of pistachio oil. Food Chem..

[7] Štampar F., Solar A., Hudina M., Veberic R., Colaric M. (2006). Traditional walnut liqueur—Cocktail of phenolics. Food Chem..

[8] Oliveira I., Sousa A., Ferreira I.C., Bento A.A., Estevinho L., Pereira J.A., Estevinho M.L.M.F. (2008). Total phenols, antioxidant potential and antimicrobial activity of walnut (*Juglans regia* L.) green husks. Food Chem. Toxicol..

[9] Shi B., Zhang W., Li X., Pan X. (2018). Seasonal variations of phenolic profiles and antioxidant activity of walnut (*Juglans sigillata* Dode) green husks. Int. J. Food Prop..

[10] Rahmani F., Dehganiasl M., Heidari R., Rezaee R., Darvishzadeh R. (2018). Genotype impact on antioxidant potential of hull and kernel in Persian walnut (*Juglans regia* L.). Int. Food Res. J..

[11] Wei Q., Ma X., Zhao Z., Zhang S., Liu S. (2010). Antioxidant activities and chemical profiles of pyroligneous acids from walnut shell. J. Anal. Appl. Pyrolysis.

[12] Del Pino-García R., González-Sanjosé M.L., Rivero-Pérez M.D., García-Lomillo J., Muñiz P. (2016). Total antioxidant capacity of new natural powdered seasonings after gastrointestinal and colonic digestion. Food Chem..

[13] Lao F., Giusti M.M. (2018). Extraction of purple corn (*Zea mays* L.) cob pigments and phenolic compounds using food-friendly solvents. J. Cereal Sci..

[14] Luo X., Bai R., Zhen D., Yang Z., Huang D., Mao H., Li X., Zou H., Xiang Y., Liu K. (2019). Response surface optimization of the enzyme-based ultrasound-assisted extraction of acorn tannins and their corrosion inhibition properties. Ind. Crops Prod..

[15] Gunathilake K., Ranaweera K., Rupasinghe H. (2018). Change of phenolics, carotenoids, and antioxidant capacity following simulated gastrointestinal digestion and dialysis of selected edible green leaves. Food Chem..

[16] Tagliazucchi D., Verzelloni E., Bertolini D., Conte A. (2010). In vitro bio-accessibility and antioxidant activity of grape polyphenols. Food Chem..

[17] Li X., Lin J., Gao Y., Han W., Chen D. (2012). Antioxidant activity and mechanism of Rhizoma Cimicifugae. Chem. Central J..

[18] Ahmad N., Zuo Y., Lu X., Anwar F., Hameed S. (2016). Characterization of free and conjugated phenolic compounds in fruits of selected wild plants. Food Chem..

[19] Zhang Q., Tong X., Qi B., Wang Z., Li Y., Sui X., Jiang L. (2018). Changes in antioxidant activity of Alcalase-hydrolyzed soybean hydrolysate under simulated gastrointestinal digestion and transepithelial transport. J. Funct. Foods.

[20] Zhang J., Hou X., Ahmad H., Zhang H., Zhang L., Wang T. (2014). Assessment of free radicals scavenging activity of seven natural pigments and protective effects in AAPH-challenged chicken erythrocytes. Food Chem..

[21] Liu F., Liu W., Tian S. (2014). Artificial neural network optimization of Althaea rosea seeds polysaccharides and its antioxidant activity. Int. J. Boil. Macromol..

[22] Cao C., Huang Q., Zhang B., Li C., Fu X. (2018). Physicochemical characterization and in vitro hypoglycemic activities of polysaccharides from Sargassum pallidum by microwave-assisted aqueous two-phase extraction. Int. J. Boil. Macromol..

[23] Chapdelaine P., Tremblay R.R., Dubé J.Y. (1978). P-Nitrophenol-alpha-D-glucopyranoside as substrate for measurement of maltase activity in human semen. Clin. Chem..

[24] Huang S., Ma Y., Zhang C., Cai S., Pang M. (2017). Bioaccessibility and antioxidant activity of phenolics in native and fermented Prinsepia utilis Royle seed during a simulated gastrointestinal digestion in vitro. J. Funct. Foods.

[25] Correa-Betanzo J., Allen-Vercoe E., McDonald J., Schroeter K., Corredig M., Paliyath G. (2014). Stability and biological activity of wild blueberry (*Vaccinium angustifolium*) polyphenols during simulated in vitro gastrointestinal digestion. Food Chem..

[26] Vallejo F., Gil-Izquierdo A., Pérez-Vicente A., Garcia-Viguera C. (2004). In Vitro Gastrointestinal Digestion Study of Broccoli Inflorescence Phenolic Compounds, Glucosinolates, and Vitamin C. J. Agric. Food Chem..

[27] Mosele J.I., Macià A., Romero M.-P., Motilva M.-J. (2016). Stability and metabolism of Arbutus unedo bioactive compounds (phenolics and antioxidants) under in vitro digestion and colonic fermentation. Food Chem..

[28] Li Y., Luo X., Wu C., Cao S., Zhou Y., Jie B., Cao Y., Meng H., Wu G. (2017). Comparative Transcriptome Analysis of Genes Involved in Anthocyanin Biosynthesis in Red and Green Walnut (*Juglans regia* L.). Molecules.

[29] Manach C., Williamson G., Morand C., Scalbert A., Rémésy C. (2005). Bioavailability and bioefficacy of polyphenols in humans. I. Review of 97 bioavailability studies. Am. J. Clin. Nutr..

[30] Kahle K., Kempf M., Schreier P., Scheppach W., Schrenk D., Kautenburger T., Hecker D., Huemmer W., Ackermann M., Richling E. (2011). Intestinal transit and systemic metabolism of apple polyphenols. Eur. J. Nutr..

[31] Chiang C.-J., Kadouh H., Zhou K. (2013). Phenolic compounds and antioxidant properties of gooseberry as affected by in vitro digestion. LWT.

[32] Sun L., Zhang H., Zhuang Y. (2012). Preparation of Free, Soluble Conjugate, and Insoluble-Bound Phenolic Compounds from Peels of Rambutan (*Nephelium lappaceum*) and Evaluation of Antioxidant Activities in vitro. J. Food Sci..

[33] Velderrain-Rodríguez G., Quirós-Sauceda A., Mercado-Mercado G., Ayala-Zavala J.F., Astiazarán-García H., Robles-Sánchez R.M., Wall-Medrano A., Sayago-Ayerdi S., González-Aguilar G.A. (2016). Effect of dietary fiber on the bioaccessibility of phenolic compounds of mango, papaya and pineapple fruits by an in vitro digestion model. Food Sci. Technol..

[34] Zheng G., Deng J., Wen L., You L., Zhao Z., Zhou L. (2018). Release of phenolic compounds and antioxidant capacity of Chinese hawthorn “Crataegus pinnatifida” during in vitro digestion. J. Funct. Foods.

[35] Tang X.-Z., Dong Y.-X., Wei S.-Q., Zhang X.-S., Yin Y.-P. (2010). Antioxidant Activity of Pigment Extracted from Green-Wheat-Bran. Agric. Sci. China.

[36] Heim K.E., Tagliaferro A.R., Bobilya D.J. (2002). Flavonoid antioxidants: chemistry, metabolism and structure-activity relationships. J. Nutr. Biochem..

[37] Bouayed J., Hoffmann L., Bohn T. (2011). Total phenolics, flavonoids, anthocyanins and antioxidant activity following simulated gastro-intestinal digestion and dialysis of apple varieties: Bioaccessibility and potential uptake. Food Chem..

[38] Kotásková E., Sumczynski D., Mlcek J., Valášek P. (2016). Determination of free and bound phenolics using HPLC-DAD, antioxidant activity and in vitro digestibility of Eragrostis tef. J. Food Compos. Anal..

[39] Kwon G.S., Forrest M.L. (2006). Amphiphilic block copolymer micelles for nanoscale drug delivery. Drug Dev. Res..

[40] Etxeberria U., De La Garza A.L., Campión J., Martínez J.A., I Milagro F. (2012). Antidiabetic effects of natural plant extracts via inhibition of carbohydrate hydrolysis enzymes with emphasis on pancreatic alpha amylase. Expert Opin. Ther. Targets.

[41] Fernández-Agulló A., Pereira E., Freire M.S., Valentão P., Andrade P., González-Álvarez J., Pereira J.A., Andrade P. (2013). Influence of solvent on the antioxidant and antimicrobial properties of walnut (*Juglans regia* L.) green husk extracts. Ind. Crops Prod..

[42] Panwar R., Raghuwanshi N., Srivastava A.K., Sharma A.K., Pruthi V. (2018). In-vivo sustained release of nanoencapsulated ferulic acid and its impact in induced diabetes. Mater. Sci. Eng. C.

[43] McDougall G.J., Stewart D. (2005). The inhibitory effects of berry polyphenols on digestive enzymes. BioFactors.

[44] Qin Y., Wang L., Liu Y., Zhang Q., Li Y., Wu Z. (2018). Release of phenolics compounds from Rubus idaeus L. dried fruits and seeds during simulated in vitro digestion and their bio-activities. J. Funct. Foods.

